# Selection of geographical populations suitable for artificial breeding of the Northeast China Brown Frog (*Rana dybowskii*)

**DOI:** 10.1007/s00114-025-02018-7

**Published:** 2025-09-03

**Authors:** Wanli Liu, Jiuchen Tao, Qing Yu, Tong Wu, Bojian Xing, Jing Bai, Wenge Zhao, Yufen Liu, Peng Liu

**Affiliations:** 1 College of Life Science and Technology, Harbin Normal University, Harbin, 150025 People’s Republic of China; 2 Biology Department, Harbin No. 3 High School, Harbin, 150025 People’s Republic of China; 3 Biology Department, Harbin No. 6 High School, Harbin, 150025 People’s Republic of China; 4 Key Laboratory of Biodiversity of Aquatic Organisms, Harbin Normal University, Harbin, 150025 China

**Keywords:** Artificial breeding, Morphological characteristics, MHC polymorphism, Population, *Rana dybowskii*

## Abstract

Amphibians, as a group greatly disturbed by human activities, are at increased risk of extinction. *Rana dybowskii* is an anuran species with both ecological and economic significance. Due to environmental changes and human overexploitation, it has been classified as Near-Threatened. This study integrates morphological and molecular immunological approaches to identify *R. dybowskii* populations with greater survival and disease resistance, based on 32 morphological traits and *MHC* class I and II polymorphism. Morphological results showed that compared with other populations, Yichun (YC) population had the highest fatness, the lowest IOD/HW, and the largest HW/SVL, HL/SVL, HW/HL, SL/TL. It indicates that YC population shows larger body size, wider vision and stronger jumping ability. The polymorphism of *MHC* I gene was the highest in Shangzhi (SZ) population, and the polymorphism of *MHC* II gene was the highest in YC population. Moreover, duplication, selection, and recombination occurred during evolution of *MHC* class I and II genes. Since both SZ and YC populations scored higher in this category (the variant sites, nucleotide polymorphism, amino-acid divergence/nucleotide divergence, dN/dS, Tajima’ D, etc.), they were more resistant to disease. All in all, these results indicated that YC population of the Lesser Khingan Mountains had good morphology and immune results, and *R. dybowskii* in the Lesser Khingan Mountains might be more suitable to be the original population of artificial breeding, which provided a theoretical basis for the realization of artificial breeding in the next step.

**Supplementary Information:**

The online version contains supplementary material available at 10.1007/s00114-025-02018-7.

## Introduction

Human activities have increasingly disrupted natural ecosystems, negatively impacting numerous animal species, including local extinctions (Pounds et al. 2006; Barnosky et al. 2011; Hannah 2013). Among vertebrates, amphibians are particularly vulnerable, as habitat loss due to anthropogenic activities significantly heightens their extinction risk (Cox et al. 2022). Amphibians Living in forest and rocky areas are more severely affected than other types. 663 amphibian species were classified as 'Critically Endangered' by the International Union for Conservation of Nature (IPCC 2021). While habitat loss remains a primary driver of population decline, commercial exploitation has also emerged as a major threat to many amphibians (Wang 2018). Consequently, economically valuable amphibian species require urgent conservation efforts, as wild populations continue to decline while demand for their resources escalates.

Oviductus Ranae of *Rana dybowskii* (Günther 1876) has been commonly used in Chinese medicine since ancient times (Commission CP 2020). Oviductus Ranae has been shown to enhance immunity, relieve fatigue, reduce oxidative damage, among other beneficial effects (Lou et al. 2014; Zhang et al. 2018; Yu et al. 2020). In addition, the recent acceptance of frogs as a food ingredient has added value to *R. dybowskii* (Wang 2022). Meanwhile, *R. dybowskii*, as a cold-climate amphibian inhabiting high latitude forests, is widely distributed in northeast China. As part of the local ecosystem, *R. dybowskii*'s ecological value in maintaining the stability of the ecosystem is self-evident (Zhao 2008). However, with the increase of human activities, increasingly serious environmental pollution and increasing capture demand, the population of *R. dybowskii* has sharply reduced to the Near Threatened status (Jiang et al. 2016). Hence, artificial breeding has become an important means to restore the population number and protect the wild resources at this stage (Wu et al. 2014).

In artificial breeding, morphological characteristics are often used as an important selection index. Among them, the larger size populations are better able to realize their value. First of all, because amphibians have life history characteristics such as hibernation, individuals with higher energy reserves have higher survival rates over winter (Costanzo 2019). Secondly, the sexual selection during the reproduction period, larger individuals are preferred by the opposite sex (Liu et al. 2013). Finally, with their larger heads and longer hind limbs, the anura could have an easier time avoiding predators and hunting (James and Wilson 2007; Changizi and Shimojo 2008; Monroy and Nishikawa 2009). So larger individuals may have an advantage in survival, hunting, reproduction, and so on. In addition, larger body size may result in more muscle and heavier Oviductus Ranae, bringing more economic benefits to breeders. However, in order to maintain the stability of the population, resistance to disease also becomes a consideration in captivity. As amphibians rely on aquatic environments, they are particularly susceptible to pathogens and harmful substances present in humid habitats, which can negatively impact both individual health and overall population fitness. For example, *Aeromonas hydrophila*, often gave *R. dybowskii* to red appendages disease, which causing tissue bleeding, organ disease and even death (Bian et al. 2020; Wu et al. 2022). *Batrachochytrium dendrobatidis* poses a serious threat to amphibians worldwide (Fisher et al. 2012). *Ranavirus sp.* is also a common pathogenic factor of *R. dybowskii* (Xu 2010). In conclusion, we need to select disease-resistant individuals for breeding in captivity. In previous studies, pathogen infection and biomacromolecule stimulation methods were usually used to judge the disease resistance of animals (Field et al. 2022), which usually caused irreversible damage to animals. With the development of biotechnology, molecular markers have become a feasible detection method in recent years (Bian et al. 2020).

Among them, major histocompatibility complex (*MHC*) may be one of the more suitable molecular markers for determining immune strength. Modern immunological studies have confirmed that the *MHC* plays a crucial role in the initiation and regulation of the immune response (Mcdevitt 2000). *MHC* has various genetic characteristics, including polygenotype, polymorphism, haploid inheritance, and linkage disequilibrium, that distinguish it from other eukaryotic gene systems. Among these, polymorphism is the most important genetic characteristic. It is generally believed that *MHC* genes themselves can lead to variations in gene structure through gene mutations, recombinations, and transformations, and other mechanisms (Bahr and Wilson 2012; Lau et al. 2016; Maria et al. 2017; Jeon et al. 2019). When an infected individual develops an immune response, there is an opportunity to pass on the stronger resistant mutation to the future generation, allowing the accumulation of mutations to remain in the form of alleles (Sommer 2005). In other words, the mechanism to maintain *MHC* polymorphism lies in pathogen-mediated balanced selection (Hedrick 1998; Borghans et al. 2004; Spurgin and Richardson 2010). When pathogenic microorganisms infect the host, there is a fierce confrontation between the pathogen and the host. To ensure the continuation of species, hosts tend to pass on *MHC* alleles with stronger resistance to the next generation. Over time, *MHC* polymorphisms gradually emerge and accumulate to a very high level (Bernatchez and Landry 2010; Gowane et al. 2013). Simultaneously, polymorphism can also occur through long-term natural selection, when the continuous accumulation of alleles eventually leads to a high degree of *MHC* polymorphism in different species (Talarico et al. 2021; Dearborn et al. 2022; Mancilla-Morales et al. 2022). For example, in the analysis of *Hoplobatrachus chinensis*, it was found that *MHC* class I and II genes underwent positive selection, and positive selection sites were detected in the presumed antigen-binding region (Cheng 2016b). *MHC* genes are closely related to the immune response. Thus, higher *MHC* gene polymorphism leads to stronger disease resistance in individuals (Abualrous et al. 2021). At last, *MHC* has also become one of the molecular markers to evaluate the fitness of a population (Buzan et al. 2022).

Through long-term selection and evolution, animals may develop regional phenotypic differences to adapt to their environments. Understanding these variations is crucial for conservation and breeding strategies. This study examines three geographical populations of *R. dybowskii* to assess their morphological traits and *MHC* gene polymorphism using morphological and molecular immunological analyses. By evaluating the survival potential and fitness of these populations, we aim to identify the most suitable group for artificial breeding. Our findings provide a theoretical foundation for both breeding programs and the scientific conservation of *R. dybowskii*.

## Material and methods

### Sample processing and molecular techniques

**Fig. 1:**
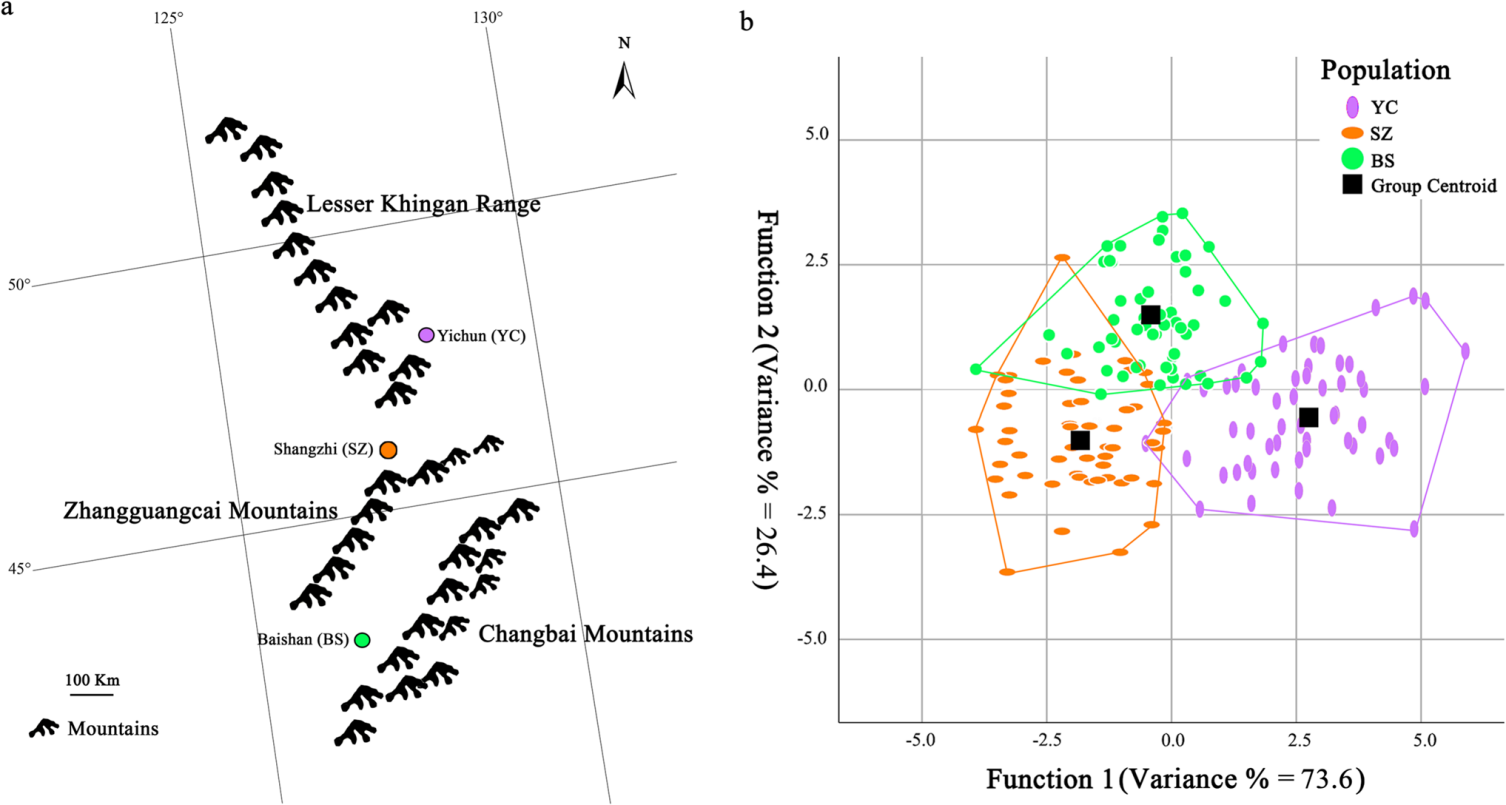
Sample map **a** and discriminant **b** of morphological characteristics of *Rana dybowskii*.** a** is a sample map of *R. dybowskii*. The population codes are as follows: YC indicates Yichun population; SZ indicates Shangzhi population; and BS indicates Baishan population

The adults *R. dybowskii* were collected in Yichun City (Heilongjiang Province, China, located in Lesser Khingan Mountains), Shangzhi City (Heilongjiang Province, China, located in Zhangguangcai Mountains), and Baishan City (Jilin Province, China, located in Changbai Mountains), and were named YC, SZ, and BS populations, respectively (Fig. 1a). More than 60 samples were used in each population (YC population: 60; SZ population: 64; BS population: 66). Institutional Animal Care and Use Committee (IACUC) of the Harbin Normal University full approval for this research (HNUARIA2020001, App date: 2020–04–01). All experiments were performed in accordance with the Regulations for the Administration of Affairs Concerning Experimental Animals, approved by the State Council of China. Morphological measurements were taken using a digital vernier calliper (Mitutoyo, precision: 0.01 mm) and an electronic balance (Mettler, precision: 0.001 g). A total of 26 morphological characteristics were recorded (Supplementary Table S1) (Zhao 2008). To further evaluate interpopulation differences in morphology, six indices related to fatness, head proportions, and limb dimensions were calculated. A full list of measured traits and calculated indices is available in Supplementary Table S1.

Statistical analyses were conducted using SPSS software v. 26.0 (SPSS, Inc., Chicago, USA). Normality and homogeneity were evaluated with the *Kolmogorov–Smirnov* test and *Levene’s* test, respectively. Data are shown as mean ± standard error (SE). The correlation between snout-vent length (SVL) and other morphological characteristics was analyzed by univariate Pearson correlation. If significant correlations were detected, analysis of covariance (ANCOVA) was conducted with SVL as a covariate. Otherwise, one-way analysis of variance (ANOVA) was applied. When multiple comparisons were performed, a Bonferroni correction was applied to control for Type I errors. The discriminant analysis was used to show the morphological differences among different populations, and principal component analysis (PCA) was used to carry out multivariate analysis on 32 morphological characteristics and indicators. The eigenvalues and eigenvectors of correlation matrix were obtained, and the contribution rate of each principal component was calculated. Significance indexes with eigenvalues greater than 1 and cumulative contribution rate of more than 60% were selected to determine the main factors. After principal component rotation, the load of each morphological feature in each principal component was obtained, so that the factor information of the response of this principal component could be inferred, and the overall characteristics of morphological indexes of different geographical populations could be better revealed.

### Sample processing and molecular techniques

The leg muscle was removed and immediately frozen in Liquid nitrogen for storage prior to study. A piece of frozen muscle weighing about 100 mg was ground into powder in liquid nitrogen. Genomic DNA was extracted using the SanPrEP column animal genomic DNA extraction kit from Sangon Biotech (Shanghai) Co., Ltd., and muscle total RNA was extracted using Takara's Trizol regent. According to *R. temporaria* sequences collected in GenBank, three pairs of specific primers were designed using Oligo 6.0 and Primer Premier 5.0 (Table 1). PCR amplifications were carried out using DNA or cDNA as templates in a 50 μL system containing 10 μL 5 × PCR buffer and 0.25 μL TaKaRa Ex Taq HS (5 U/μL) (TaKaRa, Japan), 1 μL (10 μmol/μL) forward primer, and 10 μL template. Thermal cycling program was 94 °C/2 min, (94 °C/30 s; Ta/30 s; 72 °C/30 s) for 35 cycles; 72 °C/5 min. PCR products were separated on 1.5% agarose gel. The target fragments were excised and recovered using DNA purification kit (AXYGEN, USA). After amplifying the fragment of the antigen-binding region, SSCP analysis was performed. Also, PCR products were ligated to the pMD–18 T vector and transformed into *Escherichia coli* DN5α competent cells using a pMD–18 T connection kit (TaKaRa, Japan). The competent cells were cultured for 12–16 h on LB medium containing 50 μg/mL Ampicillin. Single colonies were picked up and transferred to Liquid LB medium for culture for about 2–3 h till OD600 reached 0.5. A sample of the bacteria solution measuring 1 μL was used as template for screening 50 positive clones in the same PCR system as mentioned previously. Positive clones were sequenced using M13 +/M13–primers.

**Table 1:** Primer sequences used for *MHC*’ amplification

Primer	Sequences (5′—3′)	Annealing temperature (Ta: °C)
*MHC* I exon 3 F	gtgtaacatcttcctctactgtctgcagg	55
*MHC* I exon 3 R	tactctgtgtatgtcttcagccattctat	55
*MHC* II exon2 F	tgtgtgttgtgttctctccctg	53
*MHC* II exon2 R	ggtcaggaagggtaagagagg	53
*MHC* I-α1 + α2 F	gggtctcggataaaggat	57
*MHC* I-α1 + α2 R	tcccgtactctatgtatttct	57

### Allele determination, MHC polymorphism and its mechanism

The sequences were imported into BLAST of NCBI (http://www.ncbi.nlm.nih.gov/) for homology comparison, to determine whether the obtained sequences were *MHC* genes of *R. dybowskii*. The nucleotide and amino acid sequences identified as *MHC* genes were then compared using Clustal W in MEGA 5.0. Antigen binding sites are predicted by using related species (Bataille et al. 2015). The number of alleles was determined by deducing alleles based on the isolation characteristics of SSCP and haplotype information in Dnasp 5.0 software. Then, the sequences are deposited in GenBank.

Alleles were analyzed using Dnasp 5.0 computing nucleotide polymorphism (π) and variable sites (S). DNAMAN and MEGA were used to calculate the genetic distance and phylogenetic relationship of populations, and the population genetic variation was determined. To analyze the evolutionary relationships of *MHC* genes, this research examined the *MHC* class I and class II genes of amphibians and other vertebrates published in GenBank. MEGA software was used to construct a phylogenetic tree (Neighbor–joining model, NJ) of the *MHC* class I and *MHC* class II alleles from amino acid sequence alignment.

The nucleotide distances (Kimura 2–parameter model, K2P), amino acid distances (Poissons–corrected model), and Tajima’s D were calculated with the MEGA 5.0 software, and the bootstrap values were set to 1000. The synonymous substitution rate (dS) and non-synonymous substitution rate (dN) of antigenic binding regions, as well as the ratio of dN/dS, were obtained with MEGA 5.0, and the synonymous substitution rate (dN/dS) was determined for the Z–test. When the ratio was greater than 1, a positive selection effect was indicated. In order to calculate more accurately and eliminate software bias, MEME and Weblogo softwares were also used to detect the selection effect and verify the selected sites. Using the online software Datamonkey website (http://www.datamonkey.org) in the GARD (based on algorithm recombination detection) program, testing genes had a restructuring phenomenon.

## Results

### Morphological differences of populations

The morphological characteristics of *R. dybowskii* showed significant geographical variation. The results of multiple comparisons showed that the SVL of SZ population was significantly lower than that of the other two populations. The W of YC population was the largest, while the W of SZ population was the smallest. Five morphological traits (LFH, FAW, FL, IHMT, FL3) were significantly greater in the YC population compared to the other populations, while ten traits (SVL, IND, ULW, TM, SL, MTS, FL1, TL, FTL, MTL) were significantly larger in the BS population. SZ population was smaller than other populations except 4 morphological characteristics (IOD, EL, MTS, FTL). There were no significant differences in 11 morphological characteristics (W, HL, HW, SNL, IOD, EL, LL, MAL, BW, MTM and MTN) among the three populations (Table 2). Thus, the morphological characteristics of *R. dybowskii* were SZ < YC < BS. Significant geographical variation was also found in the analysis of morphological indicators. The multiple comparison results revealed that the YC population exhibited significantly greater values in five morphological indicators (Fatness, HW/SVL, HL/SVL, HW/HL, SL/TL) compared to the other two populations and significantly smaller values in IOD/HW (Table 3). To account for multiple comparisons and minimize Type I errors, a Bonferroni correction was applied. Consequently, the morphological indicators suggest that the YC population is the most favorable among the three.

**Table 2:** Morphological characteristics and indicators of three geographic populations of *R. dybowskii* (Length Units: mm, Weight Units: g). The asterisk indicates that this variable is significantly correlated with SVL, and SVL is used as the covariance for statistical analysis. The *P* values in bold are significant differences. Yichun population is represented by YC, Shangzhi population is represented by SZ, and Baishan population is represented by BS. The same lowercase letters in the same morphological characteristic or indicator indicate that there is no significant difference between the two populations

Morphological characteristics or indicators	YC population (n = 60)	SZ population (n = 64)	BS population (n = 66)
SVL	63.04 ± 0.35^a^ (56.83 ~ 68.18)	62.00 ± 0.86^a^ (51.87 ~ 77.48)	65.10 ± 0.83^b^ (51.01 ~ 80.06)
	*F*_2,187_ = 4.977,* P* = **0.008**		
W	27.29 ± 0.58^a^ (16.92 ~ 34.50)	22.86 ± 1.12^b^ (11.40 ~ 47.00)	25.61 ± 1.10^a^ (11.70 ~ 44.90)
	*F*_2,186_ = 2.266, *P* = 0.107*		
HL	17.74 ± 0.78^a^ (15.00 ~ 20.84)	16.84 ± 0.17^b^ (13.99 ~ 19.87)	17.40 ± 0.19^a^ (14.46 ~ 20.63)
	*F*_2,184_ = 0.398, *P* = 0.673*		
HW	20.18 ± 0.20^a^ (16.41 ~ 24.40)	18.71 ± 0.27^b^ (15.77 ~ 25.54)	20.27 ± 0.26^a^ (15.96 ~ 24.75)
	*F*_2,187_ = 1.404, *P* = 0.249*		
SNL	8.25 ± 0.08^a^ (6.74 ~ 9.84)	8.06 ± 0.09^b^ (6.97 ~ 9.74)	8.28 ± 0.10^a^ (6.66 ~ 10.39)
	*F*_2,184_ = 0.893, *P* = 0.412*		
IND	4.94 ± 0.06 (3.91 ~ 5.97)	4.90 ± 0.07 (4.10 ~ 6.29)	4.97 ± 0.05 (4.13 ~ 5.96)
	*F*_2,186_ = 5.134, *P* = 0.007*		
IOD	3.66 ± 0.08^a^ (2.46 ~ 5.01)	4.24 ± 0.07^b^ (3.00 ~ 5.31)	4.22 ± 0.07^b^ (3.10 ~ 5.47)
	*F*_2,186_ = 1.613, *P* = 0.203*		
ULW	4.10 ± 0.06^ab^ (3.01 ~ 5.10)	4.09 ± 0.05^a^ (3.31 ~ 5.08)	4.26 ± 0.08^b^ (2.54 ~ 5.58)
	*F*_2,186_ = 3.745, *P* = 0.026*		
EL	6.53 ± 0.08^a^ (5.37 ~ 7.84)	6.59 ± 0.09^a^ (5.15 ~ 8.04)	7.37 ± 0.10^b^ (5.49 ~ 9.27)
	*F*_2,187_ = 2.137, *P* = 0.122*		
TM	4.18 ± 0.07^a^ (3.33 ~ 5.41)	3.63 ± 0.08^b^ (2.23 ~ 5.03)	4.32 ± 0.07^a^ (3.22 ~ 5.69)
	*F*_2,184_ = 5.998, *P* = 0.003*		
LFH	28.16 ± 0.25^a^ (23.58 ~ 33.59)	26.62 ± 0.25^b^ (22.50 ~ 31.67)	27.96 ± 0.29^a^ (23.76 ~ 33.14)
	*F*_2,188_ = 8.803, *P* = 0.000*		
FAW	5.12 ± 0.13^a^ (3.18 ~ 7.62)	3.98 ± 0.08^b^ (2.66 ~ 5.45)	4.33 ± 0.09^c^ (2.85 ~ 5.96)
	*F*_2,186_ = 32.170, *P* = **0.000**		
LL	110.26 ± 0.89^a^ (94.91 ~ 127.89)	106.05 ± 1.15^b^ (90.94 ~ 127.28)	112.49 ± 1.41^a^ (87.26 ~ 143.84)
	*F*_2,188_ = 2.030, *P* = 0.135*		
SL	35.25 ± 0.27^a^ (30.85 ~ 40.13)	33.17 ± 0.38^b^ (27.46 ~ 40.29)	35.53 ± 0.38^a^ (28.72 ~ 42.00)
	*F*_2,187_ = 4.249, *P* = 0.016*		
FL	36.85 ± 0.34^a^ (31.12 ~ 43.38)	33.48 ± 0.37^b^ (26.62 ~ 41.43)	36.30 ± 0.42^a^ (28.09 ~ 45.12)
	*F*_2,188_ = 5.060, *P* = 0.008*		
MAL	3.74 ± 0.08 (2.33 ~ 4.83)	3.72 ± 0.06 (2.93 ~ 4.91)	3.77 ± 0.06 (2.72 ~ 5.05)
	*F*_2,184_ = 0.266, *P* = 0.767*		
BW	5.41 ± 0.07^a^ (4.33 ~ 6.63)	5.22 ± 0.08^b^ (4.10 ~ 6.96)	5.80 ± 0.09^c^ (4.07 ~ 7.11)
	*F*_2,187_ = 0.370, *P* = 0.692*		
IHMT	3.57 ± 0.05^a^ (2.82 ~ 4.37)	3.01 ± 0.07^b^ (1.9 ~ 4.29)	3.46 ± 0.04^a^ (2.73 ~ 4.26)
	*F*_2,185_ = 8.313, *P* = 0.000*		
MTM	15.55 ± 0.08^a^ (14.03 ~ 16.79)	15.10 ± 0.14^b^ (12.24 ~ 17.91)	16.14 ± 0.19^c^ (12.64 ~ 19.81)
	*F*_2,181_ = 1.685, *P* = 0.189*		
MTS	16.28 ± 0.16^a^ (13.26 ~ 19.10)	16.44 ± 0.18^a^ (14.18 ~ 20.50)	16.91 ± 0.20^b^ (13.53 ~ 21.30)
	*F*_2,186_ = 5.334, *P* = 0.006*		
MTN	4.69 ± 0.06^ab^ (3.91 ~ 5.71)	4.68 ± 0.05^a^ (3.82 ~ 5.58)	4.86 ± 0.07^b^ (3.70 ~ 6.29)
	*F*_2,183_ = 1.518, *P* = 0.223*		
FL3	10.84 ± 0.11^a^ (9.02 ~ 12.52)	10.12 ± 0.15^b^ (7.82 ~ 12.89)	10.48 ± 0.14^c^ (8.38 ~ 12.87)
	*F*_2,185_ = 4.568, *P* = 0.012*		
FL1	10.06 ± 0.19^a^ (5.93 ~ 12.69)	9.91 ± 0.12^a^ (7.94 ~ 11.97)	11.05 ± 0.15^b^ (8.88 ~ 13.44)
	*F*_2,186_ = 4.992, *P* = 0.008*		
TL	32.51 ± 0.35^a^ (26.19 ~ 38.59)	30.81 ± 0.36^b^ (25.30 ~ 36.34)	33.79 ± 0.48^a^ (25.88 ~ 43.23)
	*F*_2,187_ = 5.424, *P* = 0.005*		
FTL	29.03 ± 0.30^a^ (24.06 ~ 34.49)	29.55 ± 0.32^a^ (23.32 ~ 34.77)	30.74 ± 0.38^b^ (24.70 ~ 37.02)
	*F*_2,186_ = 3.615, *P* = 0.029*		
MTL	3.04 ± 0.06^a^ (2.12 ~ 4.13)	2.46 ± 0.05^b^ (1.65 ~ 3.23)	3.05 ± 0.06^a^ (2.13 ~ 4.15)
	*F*_2,185_ = 6.008,* P* = 0.003*		
Fatness (K)	0.011 ± 0.000^a^ (0.007 ~ 0.014)	0.009 ± 0.000^b^ (0.007 ~ 0.012)	0.009 ± 0.000^b^ (0.006 ~ 0.012)
	*F*_2,165_ = 40.353,* P* = **0.000**		
IOD/HW	0.181 ± 0.004^a^ (0.116 ~ 0.246)	0.229 ± 0.003^b^ (0.169 ~ 0.288)	0.210 ± 0.004^c^ (0.143 ~ 0.267)
	*F*_2,164_ = 38.56,* P* = 0.000*		
HW/SVL	0.320 ± 0.003^a^ (0.278 ~ 0.370)	0.302 ± 0.002^b^ (0.263 ~ 0.335)	0.310 ± 0.002^c^ (0.262 ~ 0.344)
	*F*_2,165_ = 15.541,* P* = **0.000**		
HL/SVL	0.281 ± 0.003^a^ (0.237 ~ 0.323)	0.272 ± 0.003^ab^ (0.219 ~ 0.317)	0.271 ± 0.002^b^ (0.238 ~ 0.320)
	*F*_2,164_ = 3.750,* P* = 0.026*		
HW/HL	1.146 ± 0.013^a^ (0.980 ~ 1.387)	1.116 ± 0.012^b^ (0.954 ~ 1.352)	1.143 ± 0.010^a^ (0.930 ~ 1.291)
	*F*_2,164_ = 2.694,* P* = 0.071*		
SL/TL	1.085 ± 0.010^a^ (0.966 ~ 1.259)	1.074 ± 0.005^ab^ (1.002 ~ 1.157)	1.055 ± 0.010^b^ (0.916 ~ 1.234)
	*F*_2,165_ = 3.664,* P* = **0.028**

**Table 3:** Primal factors loading capacity according to the three principal components of *R. dybowskii*

Morphological characteristics or indicators	Component			
	1	2	3	4
SL	0.893	0.048	0.008	−0.009
SVL	0.862	−0.24	−0.177	0.076
LL	0.854	−0.087	0.065	−0.055
HW	0.841	0.232	−0.309	0.121
W	0.838	0.058	−0.124	0.294
TL	0.806	−0.137	0.07	−0.43
MTM	0.789	−0.012	0.003	−0.042
FL	0.787	0.177	0.194	−0.128
LFH	0.783	0.099	0.181	−0.015
BW	0.782	−0.131	−0.05	0.084
SNL	0.777	−0.081	0.155	0.131
MTS	0.738	−0.123	−0.106	0.027
TM	0.731	0.179	−0.046	−0.011
IHMT	0.703	0.205	0.005	0.074
FL3	0.701	−0.029	0.14	−0.136
HL	0.697	0.112	0.488	0.137
FTL	0.697	−0.183	0.136	−0.15
MTN	0.653	−0.072	−0.012	0.231
FL1	0.604	−0.214	0.129	−0.052
IND	0.583	−0.09	0.136	0.32
EL	0.577	−0.082	−0.221	−0.228
MTL	0.545	0.24	0.036	−0.146
ULW	0.523	0.042	0.006	−0.219
MAL	0.382	−0.266	0.228	−0.057
HW/HL	0.341	0.184	−0.859	0.017
Fatness (K)	0.31	0.571	0.063	0.414
FAW	0.082	0.489	0.34	0.05
HW/SVL	0.054	0.715	−0.219	0.072
HL/SVL	−0.327	0.394	0.744	0.067
IOD	0.262	−0.708	−0.022	0.477
SL/TL	−0.14	0.314	−0.092	0.711
IOD/HW	−0.271	−0.782	0.154	0.364

Typical discriminant analysis of 32 morphological characteristics and indicators in three populations of *R. dybowskii* showed that individuals on the first and second discriminant axes were distributed in three regions, indicating that the three populations were significantly different from each other. According to the typical discriminant function, the discriminant accuracy of *R. dybowskii* was 89.7%. Among which, the discriminant accuracy of YC population was 90.7%, SZ population was 89.3%, and BS population was 89.1% (Fig. 1b). The results of factor analysis of 32 morphological characteristics and indicators showed that the accumulative contribution rate of the first three components reached 62.59%. The contribution rate of the first principal component was 78.13%, and the corresponding factors were SL, SVL, LL, HW, W, TL, MTM, FL, LFH, BW, SNL, MTS, TM, IHMT, FL3, HL, FTL, MTN, FL1, IND, EL, MTL, ULW, MAL, and HW/HL. The contribution rate of the second principal component was 9.38%, and the corresponding factor was fatness (K), FAW, and HW/SVL. The contribution rate of the third principal component was 3.13%, and the corresponding factor was HL/SVL. The contribution rate of the fourth principal component is 9.38%, and the corresponding factor is IOD, SL/TL, and IOD/HW (Table 3).

### Determination of alleles

**Fig. 2:**
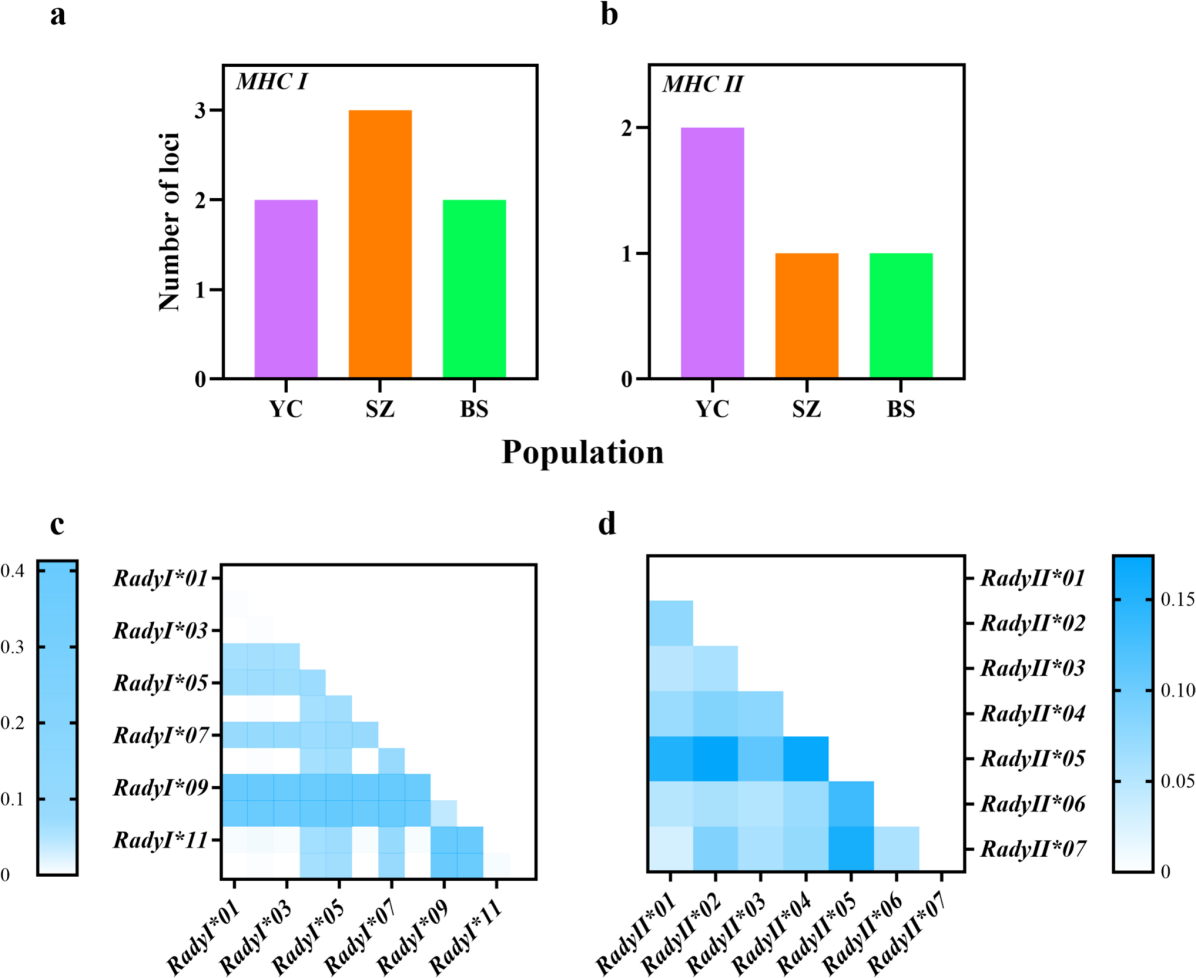
The loci and genetic distance of *R. dybowskii* in *MHC* alleles. The bar graph represents the number of loci in different populations in *MHC* I **a** and *MHC* II **b**, respectively. The genetic distance of *R. dybowskii* in *MHC* I **c** and *MHC* II **d**, respectively

A total of 300 *MHC* I and 300 *MHC* II sequences were obtained from the three *R. dybowskii* populations. The GC content of *MHC* class I genes ranged from 47.4% to 48.3%, while that of *MHC* class II genes ranged from 49.8% to 53.6%. In total, 12 *MHC* I alleles (*RadyI01*–*RadyI12*, GenBank accession numbers MW203005 − MW203016) and 7 *MHC* II alleles (*RadyII01*–*RadyII07*, GenBank accession numbers MW217991 − MW217997) were identified (Supplementary Table S2). These alleles had no intermediate terminations and could be translated into amino acids. The number of *MHC* I alleles was between 1 and 6, and the number of *MHC* II alleles was between 1 and 5 in each individual. The number of individuals with a single allele was the highest (*MHC* I: 12/30; *MHC* II: 11/30). The number of *MHC* loci possessed by different populations is shown in Fig. 2 (a, b). The SZ population (*MHC* I: 3) and YC population (*MHC* II: 2) populations had more loci than other populations. In terms of genetic distance, RadyI*09, RadyI*10 (Fig. 2c) were far away from other *MHC* Class I genes, and RadyII*05 (Fig. 2d) were far from other *MHC* Class II alleles. This was consistent with allele phylogenetic analysis (for the data see Supplementary Fig. S1a, b in Online resource). In the NJ tree of *MHC* class I and II genes, the three populations of *R. dybowskii* did not cross with other species but formed separate branches. The results also showed that the alleles of amphibian species were in a branch, while fish, mammals, and birds were separated into their own branches (for the data see Supplementary Fig. S1c, d in Online resource).

### MHC polymorphism analysis

**Fig. 3:**
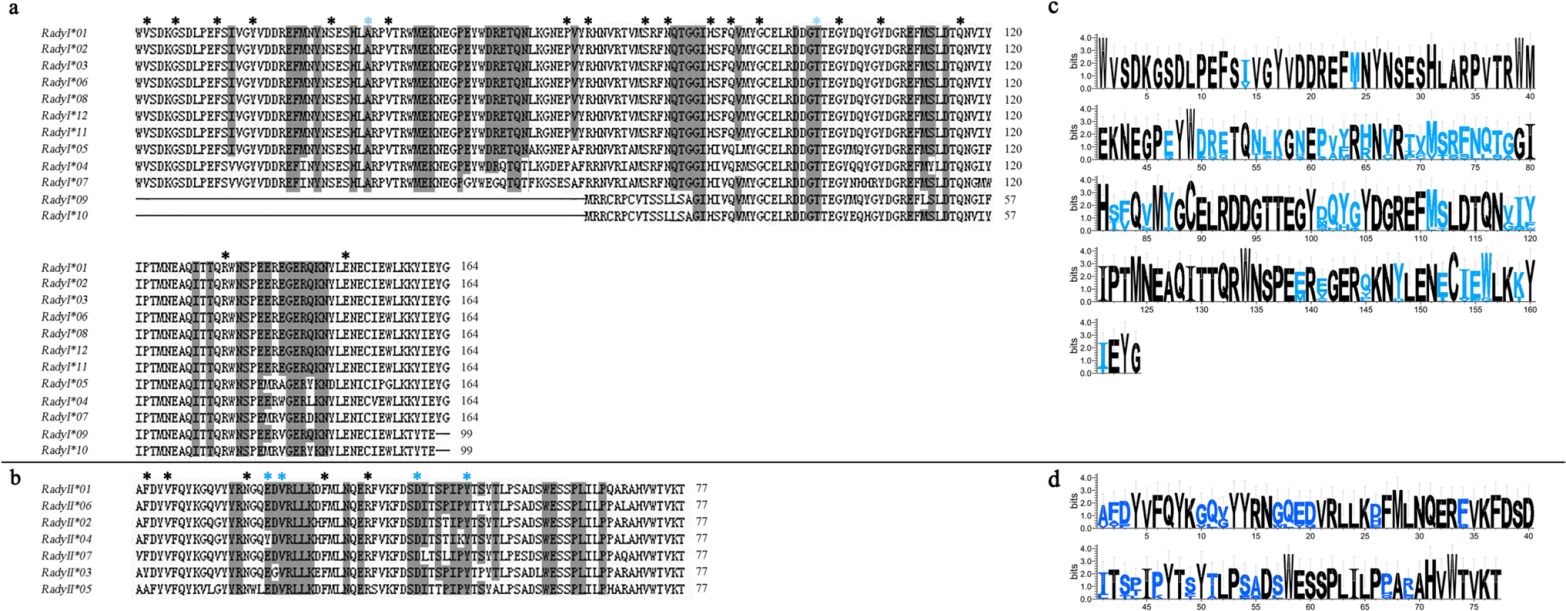
Amino acid sequences translated from the *MHC* alleles, the putative antigen presentation site, and selection of *MHC* genes in *R. dybowskii*. The putative antigen presentation site in *MHC* I **a** and *MHC* II **b**, respectively. Color represents the position of the putative antigen-binding site (pABS). The selection in *MHC* I **c** and *MHC* II **d**, respectively. Blue bases indicates positive selection at that site

In the allelic analysis, the nucleotide polymorphism rate of *MHC* I was 15.2% (76/500), and the nucleotide polymorphism (π) was 0.037. The nucleotide polymorphism rate of *MHC* II was 19.6% (49/250), and the nucleotide polymorphism (π) was 0.031 (Table 4). The 12 *MHC* I and 7 *MHC* II allele nucleotide sequences were translated into amino acids. The obtained *MHC* I sequence contained 156 amino acid residues with no terminator. Using MEGA 5.0 software and compared with the *HLA-A* allele sequence downloaded from GenBank, 20 hypothesized antigen presentation sites (PBR region) were found, including 11 hypothesized antigen presentation sites in the α1 region. It was speculated that there were nine putative antigen-binding sites in the α2 region, among which two were located at the putative antigen-binding sites (Fig. 3a). The *MHC* II alleles can also form a complete amino acid sequence of a polypeptide chain and show no termination. Compared with the related species allele sequence downloaded from GenBank, it was found that there were 26 amino acid change regions in these alleles. It was speculated that there were nine putative antigen-binding sites, among which four were located at the putative antigen-binding sites (Fig. 3b).

**Table 4:** Polymorphism statistics and sites undergoing positive selection of *MHC* genes in three populations of *R. dybowskii.* Yichun population is represented by YC, Shangzhi population is represented by SZ, and Baishan population is represented by BS

Genes	*MHC* I				*MHC* II			
Populations	YC	SZ	BS	Total	YC	SZ	BS	Total
Number of variant sites, S	37	47	19	76	20	33	26	49
Nucleotide polymorphism, π	0.018	0.04	0.013	0.037	0.043	0.042	0.074	0.031
Nucleotide divergence	0.032	0.065	0.014	0.035	0.039	0.031	0.071	0.025
Amino–acid divergence	0.033	0.075	0.033	0.071	0.075	0.054	0.118	0.051
Selection sites	14	16	22	26	17	14	10	22
Non–synonymous, dN	0.184863	0.216047	0.2705	0.393663	0.44584	0.379596	0.204135	0.276327
Synonymous, dS	0.184861	0.196956	0.241421	0.28216	0.4125	0.257975	0.184862	0.27139
(ω) dN/dS	1.000011	1.09914	1.120449	1.395176	1.080824	1.471445	1.10425	1.018496
Tajima’ D	3.970498	2.738765	3.524363	2.923549	2.807272	1.784432	1.279054	1.787096

### Positive selection and recombination detection

The results showed that the average nucleotide distance was smaller than the average amino acid distance of *R. dybowskii* (Table 4). The analysis revealed that the non-synonymous mutation rate exceeded the synonymous mutation rate (dN/dS > 1, Table 4), suggesting positive selection on *MHC* class I and II genes. All Tajima’s D > 0, indicating that genes are under balancing selection (Table 4). After that, the MEGA software detected *MHC* class I genes with 26 positive selection sites, including two sites in the inference of the antigen. *MHC* class II genes had 22 positive selection sites, and there were four antigen-binding sites (Table 4). Concurrently, MEME and Weblogo software were used to predict and analyze the structure of amino acid sequences. Larger letters of amino acids indicated stronger positive selection effects. These results confirm that the *MHC* class I and II genes of *R. dybowskii* are under strong positive selection. Specifically, selection for antigen-binding regions suggests a direct correlation between *MHC* polymorphism and disease resistance in this species.

In addition, if genes recombination occurred, and the breakpoint was detected in the sequences. In this study, traces of recombination were detected in the *MHC* I and *MHC* II alleles of three populations of *R. dybowskii*. According to the evaluation of AIC_C_, there may be single breakpoint in both *MHC* class I (Breakpoint = 248, AIC_C_ = 3179.9) and *MHC* class II genes (Breakpoint = 154, AIC_C_ = 1235.2).

## Discussion

Amphibians, as the earliest terrestrial vertebrates on Earth, play a crucial part in evolution. *R. dybowskii*, as a representative amphibian species in northeast China, plays a vital role in forest ecosystems (Zhao 1998; Li 2014). Research indicates that adult *R. dybowskii* can prey on over 30,000 pests annually, effectively performing ecological control functions and managing the infestation of larch caterpillars (Anonymous 2021). Additionally, as a keystone species in the food chain for birds and snakes, *R. dybowskii* plays a crucial role in shaping the ecological chain of forest-dwelling animals (Zhao 2008). Therefore, the stability of *R. dybowskii* populations has significant positive effects on the health of forest ecosystems and the conservation of species diversity (Wang and Ma 2011). However, given the current near-threatened status of *R. dybowskii*, the widespread recognition of the value of Oviductus Ranae, and the chaotic state of conservation management over the past decades, artificial breeding has become an appropriate approach to balance animal protection and economic interests (Zhao 2008; Cheng 2016a; Zhang and Chai 2017; Hu et al. 2022; Liao et al. 2023; Gou et al. 2025). Thus, a comprehensive analysis of the morphology and *MHC* gene polymorphism of *R. dybowskii*, so as to evaluate the disease resistance and population fitness of *R. dybowskii*, is of great significance for the conservation of *R. dybowskii*.

In the study of Anura morphology, there are several characteristics and indexes that can reflect the population situation. First, studies have demonstrated a positive correlation between frog fecundity and body size, with larger individuals exhibiting higher reproductive potential (Gibbons and McCarthy 1986). In this study, the BS population displayed the largest body size, followed by the YC population, which had the highest body weight. In terms of fatness, an indicator of body condition, the YC population had the highest K value, suggesting superior overall physiological status. Secondly, in the Anura, their strong limbs give them an advantage in reproduction and movement (Nauwelaerts et al. 2007; Liu et al. 2013). In this study, the forelimbs of YC population were developed and stronger than those of other populations (e.g., LFH, FAW), which may be related to mating reproduction (Li et al. 2015a). The body length of amphibians is positively correlated with the length of hind limbs (Wilson et al. 2000). Individuals with longer hind limbs have stronger swimming and jumping ability, and can get longer jumping distance or faster take-off speed, while individuals with shorter hind limbs are generally more suitable for crawling (James and Wilson 2007). Longer and more muscular hind limbs may represent an adaptation in *R. dybowskii* to enhance jumping ability, thereby improving survival and reproductive success (Zug 1972; Nauwelaerts et al. 2007). Foot (FL) of YC population was the longest, and this population exhibited the highest SL/TL ratio, suggesting superior jumping ability compared to the other populations. Head size is another critical trait in amphibians, influencing intraspecific competition, mate selection (Schäuble 2004; Herrel et al. 2012), and predation efficiency (Monroy and Nishikawa 2009). In this study, when evaluating head-related indices (IOD/HW, HW/SVL, HL/SVL, HW/HL), individuals from the YC population exhibited superior head morphology and a broader visual field, which may confer advantages in foraging and mating. Based on the above morphological results, the YC population is better than the other two populations in terms of survival and reproduction.

Subsequently, we analyzed the immune aspects of *R. dybowskii*. The number of *MHC* I alleles in *R. dybowskii* was higher than in *Xenopus laevis* (Bos and Waldman 2006) but lower than in *H. chinensis* (Cheng 2016b). The *MHC* II allele count was comparable to that of *H. chinensis*, with more loci than *H. chinensis* (Cheng 2016b) but fewer than *Odorrana tormota* (Li et al. 2015b). Furthermore, it showed that the *MHC* class I gene polymorphism of *R. dybowskii* was higher than that of *X. laevis* and lower than that of *H. chinensis*. These studies also exhibit many loci, explaining *MHC* gene copy number, or occur many times in evolutionary restructuring that leads to the evolution of species (Wang 2012). As *MHC* I and II molecules participate in body immunity through the induction of endogenous and exogenous antigen, respectively. Because of this, there is a positive correlation between them and the ability of amphibians to fight infection (Hughes and Nei 1990; Mcdevitt 2000; Savage et al. 2017). Hence, the *MHC* genes of *R. dybowskii* undergo gene duplication and gene deletion in the evolutionary process, which conforms to the change rule of the gene “birth-and-death” model of evolutionary patterns (Nei et al. 1997). The YC and SZ populations exhibited higher *MHC* gene polymorphism, indicating a potentially greater capacity for disease resistance. Later, when analyzing the cause of its polymorphism, some mutation sites were found in the putative antigen binding region (Fig. 3). In three populations of *MHC* class I and II genes of *R. dybowskii*, the ratio of amino acid genetic distance and nucleotide genetic distance and the ratio of non-synonymous mutations and synonymous mutations (all value > 1, Table 4) showed that *MHC* class I and II genes of *R. dybowskii* might have experienced positive selection, especially the higher positive selection of the antigen area (Fig. 3). Simultaneously, other amphibian species in different geographic population *MHC* class I and II genes also exhibit positive selection (Wang 2012; Hong 2015; Cheng 2016b; Li 2016; Cortázar-Chinarro et al. 2018; Trujillo et al. 2021). It can be inferred that pathogen-induced positive selection is one of the reasons for amphibian *MHC* gene polymorphism. In addition, Tajima’s Test of Neutrality was greater than 0, indicating that genes are under balancing selection (Table 4). This also means that populations were affected by balanced selection, which was consistent with the increased survival risk of *R. dybowskii* populations. *R. dybowskii* lives in a cold and humid environment in northeast China and is mainly invaded by pathogens, such as bacteria. The occurrence of positive selection helps the population improve disease resistance and produce more dominant descendant *MHC* gene combinations. This has been demonstrated in other amphibians, birds, and mammals (Kiemnec-Tyburczy et al. 2012; Yakubu et al. 2013; Niskanen et al. 2014; Hong 2015; Yu et al. 2015; Zeng et al. 2016; Li et al. 2017). Finally, the recombination sites near the antigen-binding region of *MHC* class I and II genes in *R. dybowskii* indicate that the recombination pattern of *R. dybowskii* is primarily exon shuffling, and genetic recombination also plays a role in maintaining the polymorphism of *R. dybowskii MHC* genes (Hellsten et al. 2010). In the field of amphibian research, although antimicrobial peptides have long dominated mainstream studies, the regulation of *MHC* gene polymorphism also holds significant biological importance for host defense (Cortazar-Chinarro et al. 2023; Fu 2024; Huang et al. 2025). Previous studies have shown that *MHC* genes in Anura (such as the families Pipidae, Dicroglossidae, Ranidae, and Rhacophoridae) commonly exhibit evolutionary characteristics like gene duplication, positive selection, and genetic recombination. Moreover, cross-species polymorphism of *MHC* genes in the family Ranidae frequently occurs (Bos and Waldman 2006; Cheng 2016b; Belasen et al. 2022; Lau et al. 2023). However, this study found no evident cross-species evolutionary phenomena in the *MHC* class I and II genes of *R. dybowskii*, suggesting that *MHC* genes, as ancient genetic elements, have undergone a series of differentiations during species evolution (LaFond et al. 2022). Whether factors beyond immune influences contribute to this phenomenon remains a subject for future research (Santos et al. 2010; Bererhi et al. 2023). In conclusion, gene duplication, selection, and recombination work together to maintain the polymorphism of *MHC* genes. From the perspective of different populations, both SZ and YC populations have high scores in the evaluation of maintaining *MHC* polymorphism, which may indicate that *MHC* gene makes SZ and YC populations have stronger resistance to disease.

Based on an integrated assessment of morphological traits and molecular immune markers, the YC population emerged as the most suitable for artificial breeding. *R. dybowskii* from the Lesser Khingan Mountains, with its superior phenotype and robust immunity, appears to be the optimal candidate for captive breeding programs. There are abundant water resources, dense forests and humid climate in the Lesser Khingan Mountains, which provide a good natural environment for the reproduction and survival of *R. dybowskii*. Because the production potential is big, the development prospect is broad, so as early as the end of the twentieth century appeared *R. dybowskii* breeding work (Kong 2021). However, compared with the whole artificial aquaculture method, the semi-artificial aquaculture method has various shortcomings (such as relatively small aquaculture scale, relatively backward aquaculture technology, and extensive aquaculture methods, etc.) (Huang and Liu 2022). In order to obtain higher economic benefits, it is suggested that breeders should not only select the wild population of *R. dybowskii* in the Lesser Khingan Mountains as the original breeding population, but also continue to use hybridization and other techniques to obtain individuals with better traits. In addition, we should vigorously promote the transformation of breeding methods, mainly in artificial culture, and try to isolate wild and cultivated populations to avoid problems such as changes in wild population structure and genetic pollution that damage the natural ecosystem. However, since *R. dybowskii* is sexual dimorphism, whether there is a difference in muscle content between male and female; How to increase the proportion of females in the population and the nutrient content of Oviductus Ranae; And then how to prevent population depression during artificial breeding will be the key issues for us to continue to consider in the future.

## Conclusion

Morphological and molecular immunological analysis of *R. dybowskii* showed that there were significant geographical differences in the morphological characteristics and *MHC* gene polymorphism of populations. The population of the Lesser Khingan Mountains was better in body shape, showing a higher fatness, a wider field of vision, and a stronger jumping ability. In term of immunity, the *MHC* of three geographical populations underwent gene duplication, recombination, and positive selection of pathogens and parasites during the evolutionary process. Meanwhile, the *MHC* polymorphism of populations was high in the Lesser Khingan Mountains and Zhangguangcai Mountains. Because body size is closely related to population fitness, polymorphism of *MHC* is also significantly positively associated with disease resistance. Hence, from the point of view of individual survival and disease resistance, *R. dybowskii* population of the Lesser Khingan Mountains is more suitable for artificial breeding. Although the morphological characteristics and the genetic polymorphism of *MHC* genes of *R. dybowskii* have been preliminarily analyzed, there are still many problems to be further studied before artificial breeding.

## Supplementary Information

Below is the link to the electronic supplementary material.

Supplementary file1 (DOCX 15765 KB)

Supplementary file2 (DOCX 24 KB)

Supplementary file3 (DOCX 26 KB)

## Declarations

The manuscript submitted represents our own original work and has not been published and is not being submitted or considered for publication elsewhere.

All authors participated in the work in a substantive way and are prepared to take public responsibility for the work.

The text, illustrations, and any other materials included in the manuscript do not infringe upon any existing copyright or other rights of anyone.

The authors are agreeing that all materials submitted for evaluation (including text, figures, etc.) retains the property/copyrights of The Science of Nature. In the case of not acceptable papers, after their rejection the property of all materials will backward to the authors.

Reproduction, posting, transmission or other distribution or use of the article or any material contained therein, requires a citation to the journal The Science of Nature.
