# Supplementary material for: Selection of geographical populations suitable for artificial breeding of the Northeast China Brown Frog (Rana dybowskii)
Source: Naturwissenschaften. 2025 Sep 3;112(5):66. doi: 10.1007/s00114-025-02018-7 (PMC12408692; doi:10.1007/s00114-025-02018-7)
Supplement: Supplementary file 1 — Supplementary file1 (DOCX 15765 KB) [file 114_2025_2018_MOESM1_ESM.docx]

**Fig. S1.** Phylogenetic relationships of *MHC* class alleles amono acid among *R. dybowskii* and the vertebrate species constructed by maximum-likelihood method. The phylogenetic relationship of *R. dybowskii* in *MHC* І (a) and *MHC* ІІ (b), respectively. Phylogenetic trees in *MHC* I (c) and *MHC* II (d), respectively. Bootstrap values from 1000 iterations were indicated above the branches.
