# Supplementary material for: Selection of geographical populations suitable for artificial breeding of the Northeast China Brown Frog (Rana dybowskii)
Source: Naturwissenschaften. 2025 Sep 3;112(5):66. doi: 10.1007/s00114-025-02018-7 (PMC12408692; doi:10.1007/s00114-025-02018-7)
Supplement: Supplementary file 2 — Supplementary file2 (DOCX 24 KB) [file 114_2025_2018_MOESM2_ESM.docx]

**Table S1.** Description of Morphological characteristics and indicators

| Morphological characteristics or indicators | Description |
| --- | --- |
| W | Weight |
| SVL | Snout-vent length |
| HL | Head length |
| HW | Head width |
| SNL | Snout length |
| IND | Internasal distance |
| IOD | Interobital distance |
| ULW | Upper lid width |
| EL | Eye length |
| TM | Tympanic membrane diameter |
| LFH | Length of forearm and hand |
| FAW | Forearm width |
| LL | Leg length |
| SL | Shank length |
| FL | Foot length |
| MAL | Mouth arrises length |
| BW | Bucca width |
| IHMT | Internal hemorrhage metatarsal tubercle |
| MTM | Mouth to tympanic membrane |
| MTS | Mouth to spat |
| MTN | Mouth to nose |
| FL3 | The third finger length |
| FL1 | The first finger length |
| TL | Thigh length |
| FTL | The fourth toe length |
| MTL | Metacarpal tubercle length |
| Fatness (K) | K = (W/SVL3)*100, reflect individual fatness and growth |
| IOD/HW | Binocular parallax |
| HW/SVL | Proportion of the head |
| HL/SVL | Proportion of the head |
| HW/HL | Head shape of bodies |
| SL/TL | The length ratio of the two main moment arms of the jump lever, which is related to the jumping ability |
| W | Weight |
| SVL | Snout-vent length |
| HL | Head length |
