# Supplementary material for: Selection of geographical populations suitable for artificial breeding of the Northeast China Brown Frog (Rana dybowskii)
Source: Naturwissenschaften. 2025 Sep 3;112(5):66. doi: 10.1007/s00114-025-02018-7 (PMC12408692; doi:10.1007/s00114-025-02018-7)
Supplement: Supplementary file 3 — Supplementary file3 (DOCX 26 KB) [file 114_2025_2018_MOESM3_ESM.docx]

**Table S2.** Accretion number of MHC genes from *Rana dybowskii*

| **Type** | **Gene name** | **Accretion number** |
| --- | --- | --- |
| MHC I | *RadyI01* | MW203005 |
|  | *RadyI02* | MW203006 |
|  | *RadyI03* | MW203007 |
|  | *RadyI04* | MW203008 |
|  | *RadyI05* | MW203009 |
|  | *RadyI06* | MW203010 |
|  | *RadyI07* | MW203011 |
|  | *RadyI08* | MW203012 |
|  | *RadyI09* | MW203013 |
|  | *RadyI10* | MW203014 |
|  | *RadyI11* | MW203015 |
|  | *RadyI12* | MW203016 |
| MHC II | *RadyII01* | MW217991 |
|  | *RadyII02* | MW217992 |
|  | *RadyII03* | MW217993 |
|  | *RadyII04* | MW217994 |
|  | *RadyII05* | MW217995 |
|  | *RadyII06* | MW217996 |
|  | *RadyII07* | MW217997 |
